# Growth, Carcass Composition, Haematology and Immunity of Broilers Supplemented with Sumac Berries (*Rhus coriaria* L.) and Thyme (*Thymus vulgaris*)

**DOI:** 10.3390/ani10030513

**Published:** 2020-03-19

**Authors:** Amir Ahmadian, Alireza Seidavi, Clive J. C. Phillips

**Affiliations:** 1 Department of Animal Science, Rasht Branch, Islamic Azad University, Rasht 41335-3516, Iran; hezarmakiyan@gmail.com; 2 Centre for Animal Welfare and Ethics, School of Veterinary Science, University of Queensland, Gatton 4343, Queensland, Australia; c.phillips@uq.edu.au

**Keywords:** feed additive, poultry nutrition, sumac, supplements, thyme, weight gain

## Abstract

**Simple Summary:**

Widespread use of antibiotics is known to cause resistance in bacteria, yet they are routinely used to improve growth performance in meat chickens. We investigated two medicinal plants, thyme and sumac berries, for their ability to function as an alternative to antibiotics in the diet of broilers. The actual plants or parts of the plants have rarely been fed before as supplements, mostly their extracted oils have been used instead. In our study, they were fed at 1–3% of the diet to investigate if the effects were dependent on the dose. We found evidence of improved immune systems with both medicinal plants when the chickens were tested for resistance to Newcastle Disease and influenza. Sumac berries, and to a lesser extent thyme, reduced the fat content of the birds’ abdomen, by 62 and 41%, respectively, reflecting an observed reduction in lipids in the blood. It is concluded that these two medicinal plants offer potential to replace antibiotics in the diet of meat chickens, as well as offering benefits in reducing the fat content of the birds.

**Abstract:**

Alternatives to antibiotics as growth promoters for broilers could reduce bacterial resistance to antibiotics, while at the same time maintaining growth and improving carcass composition. We investigated the benefits of adding the medicinal plants sumac and thyme at 1, 2 or 3% of the diet for male Ross broiler chicks, with four replicates of ten birds in each treatment group and a Control. Feed intake was reduced for chickens fed the sumac supplements, and, at the two higher doses, defeathered body weight was also reduced. Abdominal fat was reduced by 41% in chickens fed thyme and 62% in those fed sumac. This reflected reduced low density lipoproteins in their blood, and in higher dose thyme treatments and all sumac treatments, reduced high density lipoproteins in blood. Apart from this, there was little effect of the supplements on carcass composition. Blood glucose was reduced in the supplemented chickens. There was evidence of higher antibody titers to Newcastle disease and influenza in supplemented chickens. It is concluded that both thyme and sumac offer potential to reduce fat content and improve disease responsiveness in broiler production systems.

## 1. Introduction

In the broiler industry, birds are often reared in confinement at high stocking densities, which can predispose them to rapid disease transmission. Potentially damaging feed additives such as antibiotics, growth promoters, and anti-coccidial drugs are routinely used to enhance growth rates, combat diseases, and reduce losses. However, the persistent use of antibiotics in the diet of broilers can cause an increase in antimicrobial resistance of human and animal bacteria [1,2]. Hence there is strong consumer demand for farm animal diets to be free from routinely administered antibiotics [3]. This has led to attempts to use medical plants as alternatives to antibiotics and artificial growth promoters, e.g., [4]. Limited studies of the effects of these supplements on broiler performance have been reported in the scientific literature. 

While the essential oil of *Thymus vulgaris* (Thymol) is known to increase the production of digestive enzymes and performance in broilers [5,6,7,8,9], limited research exists evaluating the performance of commercial broilers receiving the whole plant *Thymus vulgaris* as a dietary constituent [10,11,12,13]. One of the few studies concluded that growth performance was improved by adding thyme at 0.5–1%, but no benefits to immunocompetence were reported [11]. Another, using doses of 1–3%, found benefits to feed conversion rate, that appeared to be dose dependent [12]. In a third, supplementation at lower levels, 0.2–0.8%, produced benefits in weight gain and immune status [13]. The effects of the essential oil of thyme, thymol, are better known. It can increase both growth rates and feed conversion efficiency, as well as the antibody response to sheep red blood cells [14]. Thymol can also stimulate the immune system by increasing the activity of lymphocytes, macrophages and NK cells, as well as increasing phagocytosis and interferon synthesis [15]. Both thymol and carvacrol, the main active ingredients in thyme, have antioxidant effects [16,17]. Intestinal populations of *Lactobacillus* and *Escherichia coli* have increased in broilers supplemented with thymol (5-methyl-1–2-isopropyl phenol) and carvacrol (5-isopropyl-2-methyl phenol) [18]. However, not all researchers have found any improvement in performance of broilers [19]. Carcass traits have similarly been found by some researchers to be unresponsive to thyme oils [20,21,22]. 

Sumac (*Rhus coriaria* L.) is a shrub that is commonly seen on the roadsides in the United States, producing clusters of tart, red berries, which are eaten by quail during periods of food shortage [23]. The fruit has relatively high fiber (27% in DM) and fat contents (16.2 %), low protein (7.8% of DM), and 0.15% Ca and 0.15% P [23]. When given a range of dietary ingredients to choose from, quail select sumac at just 2–4% of the diet, which suggests some nutritional wisdom since approximately 55% of seeds pass through their gastrointestinal tract undigested. If the berries comprise more than 25% of the diet the quail will lose weight [23]. However, in broilers inclusion of sumac at lower levels (0.2–0.5% of the diet) improved intestinal morphology, feed conversion ratio, reduced both plasma cholesterol and low/very low density lipoproteins (VLDL) and increased high density lipoprotein and abdominal fat [24,25]. Furthermore, improved titers against Newcastle Disease have been recorded, as well as substitution of *E. coli* by *lactobacilli* in the gastrointestinal tract [25]. However, when sumac was included at the level of 0.5% in layers’ diet, reduced egg weight was reported [26].

The benefits of feeding medicinal plants may be dose dependent, particularly given the low concentration of active ingredients in the plants. Hence, the purpose of our study was to investigate the effects of feeding sumac fruit and thyme whole plants in the powdered form at three inclusion rates in the diet on growth, carcass and organ characteristics, and immunity and hematology parameters of broiler chicks. 

## 2. Materials and Methods

### 2.1. Ethical Considerations

All procedures used in this study were approved by the Animal Ethics Committee of the Islamic Azad University, Rasht Branch, Iran (ethical approval number 11750103942004 dated 4 January 2016), and were in accordance with the European Union Directive 2010/63/EU. 

### 2.2. Location, Birds and Management

The experiment was conducted at a commercial poultry farm (Rasht city, Pache-Kenar village, Guilan, Iran) from October to November. A total of 280 male broiler chicks of Ross 308 genotype with similar body weights (mean 44.0 ± 1.3 g) were randomly assigned to four replicates of seven treatment groups of 10 birds/group from 1–42 days of age. Growth parameters were measured on all birds. From these seven treatment groups, 1 bird/replicate was used for blood sampling, for immunity parameters a different bird was used from each replicate, and for carcass samples yet another bird was used from each replicate. The birds in the control group were given the basal diet, while those in treatment groups were given supplements as detailed below:

Treatment 1: Basal diet (control);
T1: Basal diet + thyme powder at 1% from days 29–42
T2: Basal diet + thyme powder at 2% from days 29–42
T3: Basal diet + thyme powder at 3% from days 29–42
S1: Basal diet + sumac powder at 1% from days 29–42
S2: Basal diet + sumac powder at 2% from days 29–42
S3: Basal diet + sumac powder at 3% from days 29–42

The basal diet consisted of a three-phase feeding program: starter feed from d 1–7, grower feed from d 8–24, and finisher feed from d 25–42. The ingredients and nutrient composition of the diets are shown in Table 1. Feed and water were supplied ad libitum throughout the experimental period. The diets met or exceeded Ross 308 catalogue recommendations [27,28]. Sumac fruit and thyme plants were purchased as a single batch at Rasht market, Iran, dried and ground into a powder before adding to the diet. 

The experiment was repeated four times and the total number of birds used in the study was 280 (70 × 4). The broiler groups of ten were kept in cages (1.0 × 1.0 × 0.5 m), each fitted with an individual feeder and nipple drinker. The cages were kept in a windowless, environmentally-controlled room, with room temperature initially maintained at 32 °C, declining to 22 °C, as appropriate for the age of the bird, and a light cycle of 23 hour light, 1 hour dark/day. Treatment groups were allocated to cages so that they were equally distributed in the room to minimize any cage position effect. Chickens were subjected a routine vaccination program. Briefly, the birds were vaccinated against infectious Bronchitis virus (IBV) (H120; 1st day of age), Newcastle disease virus (NDV) (8th and 21st day of age), influenza (1st day of age), and Gumboro disease (IBD071IR; 14th and 23rd day of age).

### 2.3. Growth Performance

On days 28, 35, and 42 body weight and feed intake were measured. On the same days, feed conversion ratio, average daily energy intake, gain to metabolizable energy ratio, average daily protein intake, and gain to protein intake ratio were calculated and corrected for mortality.

### 2.4. Blood Serum Parameters

At the end of the growth period (day 42), blood samples were collected from four birds per treatment (1 bird/replicate group) from the wing vein. These blood samples of 1 ml/bird were collected into EDTA tubes and then centrifuged (3,000 rpm × 10 min) at room temperature. Then, plasma was stored at −20 °C until analysis. Biochemical analyses (glucose, uric acid, total cholesterol, triglycerides, cholesterol linked to high density lipoproteins (HDL), cholesterol linked to low density lipoproteins (LDL), and HDL/LDL) were performed using standard protocols of commercial laboratory kits (Pars Azmoon Co., Tehran, Iran) [29].

### 2.5. Immune Response

Four birds per dietary treatment (1 bird/replicate group) were chosen at random for blood sample collection from the brachial vein. Blood serum was separated by centrifugation (3,000 rpm × 15 min) and antibody titers against Newcastle Disease Virus (NDV) and avian influenza (AI) measured, respectively, at 14 and 28 d after the related vaccine injection, by the hemagglutination-inhibition test [30]. For this test, in U-bottom microtiter plates, two-fold serial dilutions of heat-inactivated (at 56 °C) serum were made with phosphate-buffered saline (PBS) (0.01 mol/L; pH 7.4) for total antibody. All antibody titers were recorded as log_2_ of the highest dilution of serum that agglutinated an equal volume of a 0.5% suspension in PBS [31].

### 2.6. Carcass Measurements

At the end of the study (day 42), four birds per dietary treatment (1 bird/replicate group) were selected that were approximately the average body weight of the group. They were killed by cervical dislocation to evaluate carcass traits. The defeathered carcasses were weighed before and after removal of the head and drumsticks. Viscera and abdominal fat were then removed and the carcass yields (with the abdomen empty), and the relative weights (expressed as a percentage of the eviscerated carcass) of abdominal fat, anatomical parts (breast, drumsticks, wings, head, neck, and notarium), internal organs (heart, kidneys, and pancreas) were calculated. The relative weights of organs of importance to the immune system (thymus, liver, spleen, and bursa of Fabricius) were also determined.

### 2.7. Statistical Analysis

Cages were the experimental unit for performance traits, while the individual bird was the experimental unit for carcass and organ characteristics, and immunity and hematological traits. The model assumptions of normality and homogeneity of variance were tested using Shapiro Wilk and Levene tests, respectively. Data were analyzed by the ANOVA option of the general linear model of SAS/STAT software (SAS Institute Inc., Cary, NC, USA) for a completely randomized design with dietary additive as the main effects. The statistical model used was:

Y_ijk_ = μ + T_i_ + R_ij_ + ɛ_ijk_ (1)

where: Y_ijk_ = response variables from each individual replication or pen, µ = the overall mean, T_i_ = the effect of dietary additive, R_ij_ = the inter-experimental unit (replications) error term, ɛ_ijk_ = the intra-experimental unit error term. 

Means were compared for significant differences using the LSMEANS option of SAS version 8 (SAS Institute Inc., Cary, NC, USA). Statistical significance was established at *p* ≤ 0.05.

## 3. Results

### 3.1. Feed Intake, Growth, and Body Composition

Feed intakes were reduced in T2 and S1, S2 and S3 treatments compared with the Control treatment (Table 2). Weight gain, feed conversion ratio (Table 2), and live body weight (Table 3) were not affected by treatment. Defeathered body weight was reduced for the two higher sumac feeding levels (S2 and S3) (Table 3). Carcass weight was reduced for S2 compared with S1. The abdominal fat was reduced in the thyme treatments and even more reduced in the sumac treatments, with no differences due to level of supplement feeding (Table 4). Heart, neck (Table 4), and testes (Table 5) weights were not affected by treatment, nor were the weights of economically valuable parts—breast, drumstick, and wings—or the less valuable parts—head and lungs (Appendix A Table A1 and Table A2). Relative kidney weight increased in S3 compared with S1 and the Control treatments (Table 5). 

### 3.2. Haematology and Response to Vaccination

Blood glucose concentration was higher in the Control treatment, compared with the 2 and 3% thyme treatments and the 1 and 3% sumac treatments. Total cholesterol was reduced in the sumac treatments compared with the Control (Table 6). Compared with the Control, T1 and T2, the treatments T3 and S1, S2, and S3 all had reduced triglycerides. Similarly, compared with the Control, the treatments T2, 3 and S1, S2 and S3 all had reduced high density lipoproteins and all treatments had reduced low-density lipoproteins. 

Compared with the Control, all T treatments and S2 had higher Newcastle disease antibody titers, and T3 and S3 had higher influenza antibody titers (Table 7).

## 4. Discussion 

### 4.1. Feed Intake and Growth

Feed intake was reduced by including sumac in the diet at any level, yet only the two higher sumac feeding rates reduced body weight. Previous studies have found reduced body weight at higher feeding rates than in this study [23]. Other studies with poultry have found reduced egg weight at relatively low levels [26], confirming reduced production in chickens. Thyme also reduced feed intake in our study, but only at 2%. Conversely Zhu et al. [32] found no effect of thyme oil on the feed intake of Luhua chickens, but the growth rate of the chickens was increased when adding thyme oil. Kalantar et al. [33] reported improvements in feed efficiency with thyme, including benefits in dressing weight. Zhu et al. failed to find any effect on dressing % [32]. 

### 4.2. Body Composition and Haematology

The abdominal fat weight was affected primarily by the supplements, regardless of dose, being reduced by 41% for chickens fed the thyme supplements and 62% for chickens fed the sumac supplements. This reflects reduced low density lipoproteins in the blood in all supplemented chickens, compared with the control group. Aghazadeh et al. have found reductions in total and LDL cholesterol in chickens supplemented with thyme extract [34]. We also found reduced HDL in treatments T2 and T3, in contrast to Aghazadeh et al. who did not find any effect of thyme extract on HDL. The reduction in circulating triglycerides in T3 and S1, 2 and 3 is almost certainly connected with reduced abdominal fat deposits and was also found by Aghazadeh et al. [34]. Circulating plasma lipids are responsible for nearly all of the fatty acids in adipose tissue and de novo synthesis of fatty acids is limited. The latter is enhanced by insulin and reduced by thyroxine and glucagon. The jejunum is the main site of lipid absorption in chickens. It is likely that the reduction in abdominal fat and circulating triglycerides was due to the inhibition of hepatic 3-hydroxy-3-methyle glutaryl coenzyme A (HMG-CoA) reductase activity that thyme produces. This is a key regulatory enzyme in cholesterol synthesis. Thyme also has saponins, which complex with cholesterol and prevents its absorption. In our study thyme also reduced blood glucose, whereas in other research it has increased it, which was believed to occur for the support of gluconeogenesis [35].

### 4.3. Responses to Vaccination

Compared with the Control, the response to vaccination appeared dependent on concentration, with, at least for response to the avian influenza vaccination, improved titres at the higher concentrations of inclusion of thyme and sumac. Other researchers have noted that thyme, either alone or in combination with other herbs, produced no change in response to vaccination for Newcastle Disease, but low levels of suplementation were used [11]. A multiple herbal supplement containing thyme, oregano, chamomile, and peppermint essential oils has improved vaccine titers against avian influenza and Newcastle disease [36], and another supplement with thyme, cinnamon, and turmeric has shown similar responses in titer in response to Newcastle Disease vaccination [6]. However, in such studies it is not possible to determine which herbs were causing the response. According to the review of Papatsiros et al. [37], thymol and oregano both have medium antimicrobial activity, but cinnamon has strong activity.

### 4.4. Limitations of the Study

It is uncertain whether we used the supplements for the right length of time. Most previous work used thyme or sumac from day 1–42 of age, but there is significant cost to their inclusion for such a long period of time. Therefore, we reduced the feeding period to days 29–42 of age, in the expectation that we could get the same effect on carcass quality but at reduced cost. This appears to have been the case from our results. Further research should investigate the minimum time period to get the maximum benefit.

A second limitation is that we measured only titers in response to vaccinations, not the diseases themselves. Testing our results to examine whether disease prevalence might be reduced in farms not normally vaccinating would be useful, but this could only be done over a long period, during which time there might be an outbreak.

## 5. Conclusions

Both thyme and sumac berries had benefits in reducing fat deposition in growing broiler chickens, particularly the latter. Although much less is known about the benefits of sumac than thyme, for many hematological parameters, for example triglyceride and total cholesterol concentrations in the blood, it appeared to be more potent. However, thyme appeared to be more potent than sumac against Newcastle disease, although both had similar activity against influenza. 

## Figures and Tables

**Table 1:** Feed ingredients and nutrient content of the starter (day 1–14), grower (day 15–28), and finisher (day 29–42) diets.

Ingredients (%)	Starter (d 1–7)	Grower (d 8–24)	Finisher (d 25–42)
Corn	55.6	58.6	61.6
Soybean meal (44% P)	37.10	36.2	33.5
Soybean oil	2.85	1.4	1.5
Calcium Carbonate	1.35	0.8	0.9
Gluten meal	0.98	0.65	0.16
Dicalcium Phosphate	1.25	1.30	1.15
NaCl	0.20	0.25	0.32
Mineral and Vitamin premix *	0.50	0.50	0.50
DL-Methionine	0.13	0.25	0.30
L-Lysine hydrochloride	0.04	0.05	0.07
**Total**	**100**	**100**	**100**
**Nutrient Composition**			
Energy (ME) (kcal/kg)	3000	2950	3000
Crude Protein (%)	22.5	21.0	20.0
Calcium (%)	0.98	0.95	0.90
Available Phosphorus (%)	0.49	0.47	0.45
Sodium (%)	0.16	0.17	0.15
Chloride (%)	0.18	0.18	0.17
Lysine (Total) (%)	1.27	1.12	1.05
Methionine (%)	0.60	0.48	0.45
Methionine + Cysteine (%)	0.94	0.80	0.75
Threonine (%)	0.83	0.74	0.70

* Calcium Pantothenate: 4 mg/g; niacin: 15 mg/g; vitamin B6: 13 mg/g; Cu: 3 mg/g; Zn: 15 mg/g; Mn: 20 mg/g; Fe: 10 mg/g; K: 0.3 mg/g; vitamin A: 5000 IU/g; vitamin D3: 500 IU/g; vitamin E: 3 mg/g; vitamin K3: 1.5 mg/g; vitamin B2: 1 mg/g.

**Table 2:** Performance mean (±SEM) of Ross 308 broilers at finisher period (29–42 days) of age fed diets containing 0, 1, 2, and 3% thyme (T3) and sumac in the diet powders from days 29–42.

		Feed Intake (g/d)	Weight Gain (g/d)	Feed Conversion Ratio (g/g) ^†^	Energy Intake (kcal/d)	Energy Efficiency (kcal/g) ^‡^	Protein Intake (g/d)	Protein Efficiency (g/g) ^‡^
Treatment								
**Control**		187.6^a*^	86.5	2.17	591^a^	6.85	39.4^a^	0.46
**1% Thyme (T1)**		189.0^a^	88.6	2.14	595^a^	6.73	39.7^a^	0.45
**2% Thyme (T2)**		176.8^c^	85.6	2.07	557^c^	6.51	37.1^c^	0.43
**3% Thyme (T3)**		184.1^ab^	88.0	2.09	580^ab^	6.59	38.7^bc^	0.44
**1% Sumac (S1)**		176.8^c^	85.2	2.08	557^c^	6.54	37.1^c^	0.43
**2% Sumac (S2)**		179.1^bc^	84.6	2.12	564^bc^	6.67	37.6^bc^	0.44
**3% Sumac (S3)**		175.4^c^	86.4	2.04	553^c^	6.42	36.8^c^	0.43
**Standard Error of Mean**		5.96	0.37	0.92	5.96	0.92	5.96	0.922
***p*-Value**		0.001	0.89	0.50	0.001	0.50	0.001	0.50

^*^ In columns with superscripts, means without a common superscript differ significantly at *p* < 0.05; **^†^** feed intake/weight gain; **^‡^** energy or protein intake/weight gain.

**Table 3:** Mean (±SEM) of economically relevant carcass characteristics at 42 days of age in Ross 308 broilers fed diets containing 0, 1, 2, and 3% thyme (T3) and sumac from days 29–42.

		Live Body Weight (g)	Defeathered Body Weight (g)	Carcass Weight (Full Abdomen) (g)	Carcass Weight (Empty Abdomen) (g)
Treatment					
**Control**		2517	2292^abc*^	2015^ab^	1777^ab^
**1% Thyme (T1)**		2546	2348^ab^	2070^ab^	1718^ab^
**2% Thyme (T2)**		2567	2384^ab^	2136^a^	1818^ab^
**3% Thyme (T3)**		2522	2294^abc^	2026^ab^	1736^ab^
**1% Sumac (S1)**		2566	2459^a^	2141^a^	1879^a^
**2% Sumac (S2)**		2467	2160^c^	1863^b^	1694^b^
**3% Sumac (S3)**		2406	2260^bc^	1923^ab^	1737^ab^
**Standard Error of Mean**		59.3	57.8	77.2	54.7
***p*-Value**		0.47	0.04	0.14	0.27

^*^ In columns with superscripts, means without a common superscript differ significantly at *p* < 0.05.

**Table 4:** Abdominal fat, heart, and neck actual and relative weights at 42 days of age in Ross 308 broilers fed diets containing 0, 1, 2, and 3% thyme (T3) and sumac from days 29–42.

		Abdominal Fat Weight (g)	Relative Weight of Abdominal Fat (%)	Heart Weight (g)	Relative Weight of Heart (%)	Neck Weight (g)	Relative Weight of Neck (%)
Treatment							
**Control**		41.8^a*^	1.66^a^	14.1	0.56	66.3	2.63
**1% Thyme (T1)**		25.5^b^	1.00^bc^	15.0	0.59	65.2	2.55
**2% Thyme (T2)**		26.7^b^	1.04^b^	14.8	0.57	69.5	2.71
**3% Thyme (T3)**		23.5^bc^	0.93^bcd^	18.4	0.73	65.6	2.58
**1% Sumac (S1)**		15.7^c^	0.62^d^	17.9	0.70	60.7	2.36
**2% Sumac (S2)**		15.2^c^	0.62^d^	16.2	0.66	55.3	2.24
**3% Sumac (S3)**		16.7^c^	0.69^cd^	29.5	1.26	60.0	2.51
**Standard Error of Mean**		2.64	0.107	4.98	0.226	6.26	0.240
***p*-Value**		<0.001	<0.001	0.38	0.36	0.74	0.84

^*^ In columns with superscripts, means without a common superscript differ significantly at *p* < 0.05.

**Table 5:** Testes and kidney weights at 42 days of age in Ross 308 broilers fed diets containing 0, 1, 2, and 3% thyme (T3) and sumac from days 29–42.

		Testes Weight (g)	Relative Weight of Testes (%)	Kidneys Weight (g)	Relative Weight of Kidneys (%)
Treatment					
**Control**		1.28	0.050	13.3^ab*^	0.53^b^
**1% Thyme (T1)**		1.53	0.059	14.5^ab^	0.57^ab^
**2% Thyme (T2)**		1.62	0.062	14.6^ab^	0.57^ab^
**3% Thyme (T3)**		1.24	0.049	18.1^ab^	0.72^ab^
**1% Sumac (S1)**		1.63	0.063	13.0^b^	0.50^b^
**2% Sumac (S2)**		1.55	0.063	15.2^ab^	0.62^ab^
**3% Sumac (S3)**		1.39	0.058	18.4^a^	0.77^a^
**Standard Error of Mean**		0.207	0.008	1.58	0.066
***p*-Value**		0.75	0.78	0.13	0.087

^*^ In columns with superscripts, means without a common superscript differ significantly at *p* < 0.05.

**Table 6:** Blood parameters of Ross 308 broilers at 42 days of age fed diets containing 0, 1, 2, and 3% thyme (T3) and sumac from days 29–42.

		Glucose (mg/dL)	Uric acid (mg/dL)	Total cholesterol (mg/dL)	Triglycerides (mg/dL)	HDL Cholesterol (High Density Lipoproteins) (mg/dL)	LDL Cholesterol (Low Density Lipoproteins) (mg/dL)	LDL/HDL
Treatment								
**Control**		186.3^a^	3.85	174^a^	130^a^	91.8^a^	78.3^a^	0.86^a^
**1% Thyme (T1)**		160.5^ab^	3.05	152^abc^	126^a^	79.8^ab^	51.0^b^	0.64^ab^
**2% Thyme (T2)**		137.7^b^	2.87	158^ab^	118^a^	67.5^b^	52.2^b^	0.77^ab^
**3% Thyme (T3)**		129.0^b^	2.90	138^bcd^	58^b^	75.3^b^	51.5^b^	0.71^ab^
**1% Sumac (S1)**		136.0^b^	2.80	121^d^	60^b^	69.5^b^	40.2^b^	0.58^ab^
**2% Sumac (S2)**		153.7^ab^	3.00	128^cd^	41^b^	68.5^b^	39.0^b^	0.58^ab^
**3% Sumac (S3)**		139.7^b^	3.72	133^cd^	47^b^	73.0^b^	30.0^b^	0.43^b^
**Standard Error of Mean**		11.52	0.606	7.85	11.2	5.09	7.44	0.107
***p*-Value**		0.03	0.80	0.001	<0.001	0.04	0.005	0.15

^*^ In columns with superscripts, means without a common superscript differ significantly at *p* < 0.05.

**Table 7:** Immune response mean (±SEM) after vaccination (influenza and Newcastle) of Ross 308 broilers fed diets containing 0, 1, 2, and 3% thyme (T3) and sumac from days 29–42.

		Antibody Titer Against Last Injection of Newcastle Disease (lg2)	Antibody Titer Against Avian Influenza (lg2)
Treatment			
**Control**		3.25^c^	2.50^c^
**1% Thyme (T1)**		5.50^ab^	2.50^c^
**2% Thyme (T2)**		6.00^ab^	3.00^bc^
**3% Thyme (T3)**		7.25^a^	4.75^a^
**1% Sumac (S1)**		4.75^bc^	4.00^abc^
**2% Sumac (S2)**		5.75^ab^	6.00^bc^
**3% Sumac (S3)**		4.75^bc^	5.75^ab^
**Standard Error of Mean**		4.252	3.474
***p*-Value**		0.006	0.01

^*^ In columns with superscripts, means without a common superscript differ significantly at *p* < 0.05.

## Appendix A

**Table A1:** Mean (±SEM) of economically valuable parts in relation to eviscerated carcass at 42nd days of age in Ross 308 broilers fed diets containing 0, 1, 2 and 3% Thyme (T3) and sumac from days 29–42.

		Breast Weight (g)	Relative Weight of Breast (%)	Drumsticks (Thighs) Weight (g)	Relative Weight of Drumsticks (Thighs) (%)	Wings Weight (g)	Relative Weight of Wings (%)
Treatment							
**Control**		587	23.4	742	29.5	181	7.2
**1% Thyme (T1)**		561	22.0	726	28.6	193	7.6
**2% Thyme (T2)**		575	22.4	779	30.3	167	6.5
**3% Thyme (T3)**		569	22.6	753	29.9	160	6.4
**1% Sumac (S1)**		586	22.9	724	28.3	147	5.8
**2% Sumac (S2)**		545	22.1	643	25.9	175	7.1
**3% Sumac (S3)**		551	23.0	723	30.1	172	7.2
**Standard Error of Mean**		19.4	0.80	46.0	1.87	19.8	0.82
***p*-Value**		0.65	0.90	0.55	0.66	0.77	0.74

**Table A2:** Mean (±SEM) of less valuable body parts at 42nd days of age in Ross 308 broilers fed diets containing 0, 1, 2 and 3% Thyme (T3) and sumac from days 29-42.

	Trait	Head Weight (g)	Relative Weight of Head (%)	Lungs Weight (g)	Relative Weight of Lungs (%)
Treatment					
**Control**		69.5	2.76	9.22	0.36
**1% Thyme (T1)**		73.5	2.90	9.43	0.36
**2% Thyme (T2)**		67.5	2.63	10.50	0.41
**3% Thyme (T3)**		69.5	2.76	8.59	0.34
**1% Sumac (S1)**		67.8	2.64	6.99	0.27
**2% Sumac (S2)**		70.3	2.85	9.83	0.40
**3% Sumac (S3)**		73.8	3.07	8.88	0.37
**Standard Error of Mean**		3.46	0.162	1.701	0.067
***p*-Value**		0.78	0.51	0.86	0.84
